# Adventure and mental health: an ecological perspective

**DOI:** 10.3389/fpsyg.2024.1352352

**Published:** 2024-08-30

**Authors:** Eric Brymer, Vinathe Sharma-Brymer, Royce Willis, Matthew Leach

**Affiliations:** ^1^Faculty of Health, Southern Cross University, Gold Coast, QLD, Australia; ^2^Manna Institute, New England University, Lismore, NSW, Australia; ^3^School of Law and Society, University of the Sunshine Coast, Maroochydore, QLD, Australia

**Keywords:** adventure, mental health, ecological psychology, nature, spiritual, transformation, affordances, wellbeing

## Abstract

In this paper, we explore the concept of outdoor adventure in the context of mental health, proposing an ecological perspective to redefine adventure beyond its traditional associations with risk and physical challenge. We critically examine current frameworks and definitions of adventure, which often emphasise specific activities and natural environments yet overlook the individual’s experience and potential mental health benefits. From an ecological perspective, we emphasise the dynamic interplay between the individual, the activity, and the environment. This approach, grounded in ecological psychology, considers the multi-dimensional nature of adventure, including social, psychological, physical, and spiritual aspects. The paper challenges the traditional risk-focused view of adventure, advocating for a broader definition that includes diverse experiences and interactions, as well as the transformative potential of adventure in various environments, including urban settings. We highlight the changing demographics and motivations of adventure participants, moving away from the stereotypical young male risk-seeker, to more diverse and inclusive participant profiles. We argue that adventure facilitates the realisation of fundamental human affordances, often untapped in daily life, thereby enhancing mental health and wellbeing. This ecological perspective opens new avenues for understanding the role of adventure in mental health and wellbeing, offering a more comprehensive and inclusive approach to adventure activities as therapeutic interventions. This redefined understanding of adventure emphasises its potential as a powerful tool for enhancing human wellbeing, harmonising the relationship between people, tasks, and the environment, and offering profound implications for mental health contexts.

## Introduction

In the evolving landscape of mental health interventions, the role of outdoor adventure has garnered increasing attention for its potential therapeutic benefits. In this paper, we explore and redefine the concept of adventure in a mental health context, moving beyond traditional perspectives to a more inclusive and comprehensive understanding. The intersection of outdoor adventure and mental health presents a unique opportunity to examine how diverse adventure activities, varying from bushwalking to BASE jumping, contribute to mental wellbeing.

Traditionally associated with risk and physical challenge (Brymer, 2010) adventure is now being re-examined through a broader lens encompassing various experiences and participant demographics (Immonen et al., 2022). We seek to dissect and reconstruct the conceptual framework of adventure, challenging the conventional notion that it is solely the domain of young, risk-seeking individuals. Our discussion extends to a diverse range of participants, acknowledging that mental health benefits derived from adventure are not confined to a specific age group, gender, or cultural background.

In establishing a new theoretical framework, we delve into the definitions and frameworks of adventure, scrutinising their limitations and proposing an ecological perspective emphasising the dynamic interplay between the individual, the activity, and the environment. This perspective recognises the multi-dimensional nature of adventure and its potential to foster mental health and wellbeing.

Through this exploration, we address several key areas: the characteristics of adventure activities and their perceived risks, the role of the environment in adventure experiences, and the diverse characteristics and lived experiences of adventure participants. By integrating these elements, we aim to present a more holistic view of how adventure could be a powerful tool for improving mental health and wellbeing, transcending traditional boundaries and offering new pathways for therapeutic interventions.

We challenge existing paradigms and introduce a novel framework for understanding the complex and multifaceted relationship between adventure and mental health. By reconceptualising this relationship, we open doors to more inclusive, effective, and varied therapeutic approaches that recognise the full spectrum of human experience in adventure.

## A brief overview of adventure

Despite its prevalence in therapeutic contexts, the concept of adventure lacks a precise definition, a challenge consistently highlighted in the literature (Beard et al., 2003; Buckley, 2006; Buckley, 2021). This ambiguity arises partly from the diverse nature of activities labelled as adventure, ranging from kayaking and bushwalking to more inherently dangerous endeavours (such as BASE jumping). Current frameworks often encapsulate adventure within risk, engagement with nature, and challenge, yet these elements are variably interpreted and applied (Brymer, 2010; Immonen et al., 2022).

In therapeutic settings, adventure therapy often combines various outdoor activities, with the underlying assumption that these engagements, despite their nebulous definitions, offer risk and cognitive, kinaesthetic, and emotional stimulation for participants (Brymer and Feletti, 2020; Russell et al., 2020). This approach, while beneficial, tends to overlook the nuanced differences between various forms of adventure and their specific impacts on mental health.

Adventure presents itself as a multi-dimensional construct. However, existing frameworks, while helpful, tend to oversimplify the adventure experience, often reducing it to a set of activities or environmental settings (Peacock et al., 2017). This reductive approach risks omitting the deeper, more intrinsic values and experiences that adventure can offer, especially in the context of mental health and wellbeing.

It is evident that present frameworks, while foundational, are not sufficiently equipped to address the complex and individualised nature of adventure and its therapeutic potential. We therefore seek to explore beyond these conventional boundaries, aiming to develop a more comprehensive and inclusive framework that acknowledges the varied dimensions of adventure and its role in mental health promotion.

### Characteristics of adventure activity

Adventure, in its broadest sense, is most often associated with engagement in physical activities (mostly in outdoor settings) that encompass an element of risk (Immonen et al., 2022). Activities are diverse, spanning terrestrial pursuits like climbing, abseiling, caving, skiing, and mountain biking; aquatic activities such as diving, snorkelling, whitewater rafting, and kayaking; and aerial endeavours like BASE jumping, skydiing, and ballooning to name but a few. Beard et al. (2003) suggests that these activities form a continuum from *mild* or *soft* adventure to *hard* or *extreme* adventure, differentiated by varying ‘degrees of challenge, uncertainty, setting familiarity, personal abilities, intensity, duration and perceptions of control’ (p. 32). Mental health benefits have been reported across this spectrum of activity, underscoring the potential therapeutic value of these varied experiences.

The emphasis on risk and uncertainty as core to adventure poses challenges and raises critical questions, particularly in therapeutic contexts. Risk perception is highly subjective; an activity deemed risky and exhilarating by one individual might be viewed as mundane by another (Peacock et al., 2017). This variability in perception challenges the appropriateness of a one-size-fits-all approach in adventure-based therapy, where the willingness to engage in potentially risky activities is a prerequisite. The traditional notion which assumes risk is central to adventure has been challenged in recent years with critics pointing out that the role of risk questionable and the risk metaphor might even have damaging limitations (Bell, 2017; Brown and Fraser, 2009; Brymer, 2010; Immonen et al., 2022; King et al., 2020). The dominant focus on risk in the literature has led to a paradoxical and narrow definition of adventure, often overlooking lived experience and other valuable aspects and experiences contributing to mental health (Boudreau et al., 2022). For example, Pomfret et al. (2023) suggested that adventure impacts wellbeing through extraordinary experiences, transformative experiences, immersion and community (Pomfret et al., 2023).

Criticisms of traditional definitions of adventure point to the need for a more nuanced and richer conceptualisation of adventure that acknowledges its multifaceted nature. Adventure is not merely about the thrill and challenge of risk-taking; it encompasses a range of experiences and interactions with the environment and oneself. Recognising the diverse motivations and outcomes associated with different adventure activities is crucial to understanding the role of these activities in promoting mental health and wellbeing.

We call for a broader definition of adventure that looks beyond the conventional risk-centric view. By embracing the diverse dimensions of adventure (including the emotional, cognitive, spiritual and environmental aspects), we will be able to better understand and harness the therapeutic potential of adventure activities. Such a comprehensive approach acknowledges each individual’s unique experiences and the transformative possibilities that adventure can offer in the context of mental health.

### Characteristics of the environment

The role of the environment in adventure activities has been a central focus in defining and understanding adventure. Definitions often emphasise the concept of being *place-based*, with a predominant focus on remote and natural settings as the ideal locales for adventure activities (Dobud et al., 2020; Pomfret et al., 2023). This view posits that the quintessential adventure experience is deeply intertwined with dynamic natural environments (including wilderness areas), often unfamiliar and distinct from the participant’s regular surroundings.

This traditional emphasis on remote and natural settings as essential for adventure has faced criticism for being overly restrictive. Some authorities have argued for a broader interpretation of adventure environments, perhaps a continuum of environments, encompassing remote wilderness, rural, regional, near urban and urban settings (Sharma-Brymer et al., 2025). A systematic review by Harper et al. (2021) underscores the limited understanding of nature’s role in adventure, suggesting that the therapeutic benefits of adventure are not confined to natural settings alone. For instance, activities like urban parkour, as discussed by Clough et al. (2016), demonstrate that, while adventure in nature contexts might be ideal, mental health and wellbeing benefits can also arise from adventures in urban and indoor contexts.

The conventional view that adventure is primarily a function of the task and the environmental setting, often characterised by risk and physical challenge in natural terrains, is thus being reevaluated. Critics of this traditional perspective suggest that adventure can equally occur in everyday urban settings (Clough et al., 2016), where risk and uncertainty are managed differently, perhaps even to the point of being practically non-existent. This critique highlights the often neglected lived experience of adventure participants. As these critiques suggest, the essence of adventure should not be solely defined by the activity or the environment, but also by how individuals interact with and experience these settings.

Current discourse invites a rethinking of how we define the environment in adventure experiences. This dialogue encourages a shift from a narrow focus on remote or natural settings to a more inclusive view that acknowledges the variety of environments where adventure can occur. Understanding how different settings, whether natural or urban, contribute to mental health and wellbeing is crucial. This broader perspective allows for a more comprehensive understanding of how adventure can facilitate mental health and wellbeing in varied environmental contexts.

### Characteristics and lived experience of adventure participants

Beard et al. (2003) eloquently argue that ‘adventure is not determined by specific activities, but by the state of mind and approach of the participation’ (p. 14). This perspective marks a significant shift from other conceptualisations of adventure, which are predominantly associated with young male risk-seekers (Cater, 2013; Elsrud, 2001; Palmer, 2002; Pizam et al., 2004). However, current understandings of adventure reflect a changing demographic landscape, with growing diversity of participants (Pomfret and Bramwell, 2016). No longer can adventure be seen as the sole purview of the young; participation spans generations, with an increasingly equitable representation of genders.

Sharma-Brymer argues that the mental health benefits of adventure are accessible to individuals regardless of their marital status, geographic origin, or socio-cultural and religious affiliations. This inclusivity is significant, denoting a departure from the homogeneity that once characterised adventure participants. The motivations driving individuals towards adventure activities for mental wellbeing are diverse and complex (Kerr and Houge Mackenzie, 2012).

Adventure is characterised not only by the physical experience, but also by its capacity to undergo deep personal transformation, forge a connection and deeper relationship with nature, facilitate mindfulness and flow, enhance overall wellbeing, and offer new perspectives on life and social interactions (Boudreau et al., 2020; Brymer, 2013; Brymer and Houge MacKenzie, 2015; Clough et al., 2016; Monasterio and Brymer, 2015; Sharma-Brymer, 2022). These profound impacts often transcend physical activity to encompass religious, socio-cultural and planetary dimensions.

Naidoo et al. (2015) introduces the concept of push and pull factors in adventure, which further illuminates the motivations behind participation. Push factors, such as personal development, escapism, and social interaction, drive individuals away from their routine environments toward adventure activities. Conversely, pull factors draw individuals towards specific activities or destinations. Thus, adventure offers an escape from the mundanity of everyday life, as well as an opportunity for self-enhancement, and, for some, a safe environment to test and expand personal capabilities.

The archetypical image of the adventure participant as a young male risk-seeker is rapidly evolving. Today’s adventurers have diverse backgrounds, encompassing all genders, ages, marital statuses, and cultural contexts. Therefore, the mental health and wellbeing benefits of adventure are not limited to a specific demographic but are accessible and relevant across the lifespan, reflecting the universal appeal and transformative potential of adventure experiences.

## Towards defining adventure

The conceptualisation of adventure has predominantly revolved around two fundamental pillars: the nature of the activity, and the characteristics of the environment (Immonen et al., 2022). This perspective has typically portrayed adventure as primarily risk-focused activities in unfamiliar, most often, natural terrains. However, this narrow focus on risk and specific environmental settings has been increasingly scrutinised and critiqued (Boudreau et al., 2022). The modern conceptualisation of adventure challenges these traditional norms, suggesting that adventure need not always involve unfamiliar or exclusively natural environments.

A significant limitation of traditional definitions of adventure is their lack of emphasis on the individual’s experience. These definitions often overlook the subjective nature of adventure by primarily focusing on external factors such as risk and environment. This oversight is critical, as recent perspectives considering the individual’s experience reveal that the conventional notion of risk-taking is not universally applicable (Brymer and Schweitzer, 2013a, 2013b, 2015; Immonen et al., 2022). The adventurer, it turns out, seeks more than just risk; their motivations and experiences are far more nuanced and varied.

To address the shortcomings of traditional definitions of adventure, we propose a new theoretical conceptualisation of adventure that acknowledges the traditional pillars of activity and environment and gives equal importance to the individual’s experience. This approach recognises the potential for dynamic interactions between these three pillars: the individual, the task, and the environment. Such a framework allows for a more holistic understanding of adventure, encompassing the diverse motivations, experiences, and outcomes associated with adventure activities.

This conceptualisation underscores the need to explore how interactions between the individual, task, and environment contribute to mental health and wellbeing benefits. It is imperative to understand that adventure is not a one-dimensional concept defined solely by physical risk or environmental setting; instead, it is a multifaceted experience that can be meaningful, transformative, and beneficial in various ways to different individuals. By adopting this broader perspective, we can better appreciate the complexity of adventure and its potential role in enhancing mental health and wellbeing.

### An ecological perspective on the nexus between adventure and mental health and wellbeing

Adopting an ecological perspective shifts the focus from viewing the individual, activity, and environment as separate static components, to understanding them as dynamic, interdependent constructs. This framework, grounded in ecological psychology, has been instrumental in interpreting behaviour across various fields, including health, sport, and education (Brymer and Davids, 2013, 2014; Brymer and Davids, 2016; Brymer et al., 2014; Davids et al., 2016; Immonen et al., 2022; Sharma-Brymer et al., 2015). The framework emphasises the multi-dimensional nature of adventure, including the environment, the activity, and the interplay between the social, psychological, physical, and spiritual aspects of the individual,. This interconnectedness is crucial to understanding the adventure-mental health nexus.

Central to this ecological framework are three concepts: affordances, form of life, and effectivities (Gibson, 1979; Rietveld and Kiverstein, 2014; Stoffregen, 2003). Affordances represent the opportunities for action provided by the environment, reflecting a combination of its objective characteristics and the individual’s subjective perception. Form of life pertains to typical patterns of interaction that a group of organisms, such as humans, exhibit with their environment, manifesting as social or cultural tendencies. Effectivities refer to the individual’s skills, capacities, and capabilities that align with the affordances in the person-environment relationship.

This ecological perspective proposes that adventure offers a unique opportunity to engage with a landscape of affordances, allowing individuals to explore and realise potentials that might remain untapped in their daily lives. Adventure activities provide a rich variety of experiences essential to the human form of life, promoting adaptability and the ability to perceive and act upon a broad spectrum of affordances. This contrasts with the safety-seeking tendencies of modern life, where technological advancements and a focus on security often limit our interaction with affordances in the person-environment relationship.

In adventure settings, the person-environment relationship offers diverse affordances, such as a deeper connection with nature or opportunities for social bonding, mindfulness, flow and personal agency (Li et al., 2021; Pomfret et al., 2023). The role of adventure providers is crucial in this context. These providers design experiences that educate and guide participants, especially novices, in recognising and engaging with these affordances. This guidance helps individuals realise skills and capacities that may be underutilised in everyday life. For more experienced adventurers, their adeptness at attuning to information in the environment allows them to perceive and act on these affordances independently.

The ecological perspective redefines adventure not by the activity or the environment alone, but by the dynamic interplay between the individual, task, and environment. This perspective positions adventure as crucial in modern times, where limitations on human interactions have diminished their engagement with the wide variety of affordances available. Adventure becomes a means to rebalance this relationship, enhancing mental health and wellbeing by facilitating a rich and varied interaction with a person’s surroundings.

In essence, adventure, from an ecological standpoint, is a critical element in contemporary life. Adventure provides a platform for individuals to engage with diverse affordances, fostering personal growth and enhancing mental health and wellbeing. This redefinition of adventure underscores its unique capacity to harmonise the relationship between people, tasks, and the environment, offering profound implications for how people approach adventure in mental health contexts.

## Conclusion

Traditional perspectives on adventure have predominantly emphasised the activity and the environment, often underlining elements of risk and interaction with natural terrains. These conventional views have been challenged for their limited scope, particularly regarding their risk-centric focus and the implied necessity of natural settings. Critics have highlighted that adventure can occur in diverse environments, including urban and artificial settings. Perhaps more significant is that traditional definitions have often overlooked the vital role of the individual and the person-environment relationship in the adventure experience.

This paper proposes an alternative approach to understanding adventure, focusing on the dynamic interplay between the person and the environment. This perspective moves beyond the conventional emphasis on physical risk and environmental setting, instead highlighting the importance of an individual’s interaction with their surroundings. The approach posits that adventure, in essence, is about facilitating the realisation of fundamental human affordances. These affordances, while potentially available to all, are often underexplored or inaccessible in the routine of daily life.

By adopting an ecological perspective, we argue that adventure offers unique opportunities for individuals to engage with their environment, and to broaden their perception of human capability. This approach broadens the definition of adventure and underscores its significance in promoting mental health and wellbeing. Adventure, in this context, is not just an activity; it is a medium through which individuals can connect with a deeper sense of self and the world around them.

Redefining adventure through the lens of the person-environment relationship opens new avenues for understanding its role in mental health and wellbeing. It encourages a more inclusive and holistic approach to adventure activities, recognising the diverse ways individuals can engage with and benefit from these experiences. Ultimately, this perspective offers a more comprehensive understanding of adventure, emphasising its potential as a powerful tool for enhancing human wellbeing.

## Data Availability

The original contributions presented in the study are included in the article/supplementary material, further inquiries can be directed to the corresponding author.

## References

[ref1] Beard C. Swarbrooke J. Leckie S. Pomfret G. (2003). Adventure tourism. 1st Edn London: Routledge.

[ref2] Bell M. (2017). The romance of risk: adventure’s incorporation in risk society. J. Advent. Educ. Outdoor Learn. 17, 280–293. doi: 10.1080/14729679.2016.1263802

[ref3] Boudreau P. Mackenzie S. H. Hodge K. (2020). Flow states in adventure recreation: a systematic review and thematic synthesis. Psychol. Sport Exerc. 46:101611. doi: 10.1016/j.psychsport.2019.101611

[ref4] Boudreau P. Mackenzie S. H. Hodge K. (2022). Adventure-based mindsets helped maintain psychological well-being during COVID-19. Psychol. Sport Exerc. 62:102245. doi: 10.1016/j.psychsport.2022.102245, PMID: 35755019 PMC9212858

[ref5] Brown M. Fraser D. (2009). Re-evaluating risk and exploring educational alternatives. J. Advent. Educ. Outdoor Learn. 9, 61–77. doi: 10.1080/14729670902789529

[ref6] Brymer E. (2010). Risk taking in extreme sports: a phenomenological perspective. Ann. Leisure Res. 13, 218–238. doi: 10.1080/11745398.2010.9686845, PMID: 22689592

[ref7] Brymer E. (2013). “Extreme sports as transformational tourism” in Transformational tourism: Tourist perspectives. ed. Reisinger Y. (Wallingford, UK: CABI), 111–114.

[ref8] Brymer E. Davids K. (2013). Ecological dynamics as a theoretical framework for development of sustainable behaviours towards the environment. Environ. Educ. Res. 19, 45–63. doi: 10.1080/13504622.2012.677416

[ref9] Brymer E. Davids K. (2014). Experiential learning as a constraint-led process: an ecological dynamics perspective. J. Advent. Educ. Outdoor Learn. 14, 103–117. doi: 10.1080/14729679.2013.789353

[ref10] Brymer E. Davids K. (2016). Designing environments to enhance physical and psychological benefits of physical activity: a multidisciplinary perspective. Sports Med. 46, 925–926. doi: 10.1007/s40279-016-0535-8, PMID: 27113737

[ref11] Brymer E. Davids K. Mallabon L. (2014). Understanding the psychological health and well-being benefits of physical activity in nature: an ecological dynamics analysis. Ecopsychology 6, 189–197. doi: 10.1089/eco.2013.0110

[ref12] Brymer E. Feletti F. (2020). Beyond risk: the importance of adventure in the everyday life of young people. Ann. Leisure Res. 23, 429–446. doi: 10.1080/11745398.2019.1659837

[ref13] Brymer E. Houge MacKenzie S. (2015). “The impact of extreme sports on host communities’ psychological growth and development” in Transformational tourism: Host benefits. ed. Reisinger Y. (Wallingford, UK: CABI), 129–140.

[ref14] Brymer E. Schweitzer R. (2013a). Extreme sports are good for your health: a phenomenological understanding of fear and anxiety in extreme sport. J. Health Psychol. 18, 477–487. doi: 10.1177/1359105312446770, PMID: 22689592

[ref15] Brymer E. Schweitzer R. (2013b). The search for freedom in extreme sports: a phenomenological exploration. Psychol. Sport Exerc. 14, 865–873. doi: 10.1016/j.psychsport.2013.07.004

[ref16] Brymer E. Schweitzer R. (2015). “Phenomenology and extreme sports in natural landscapes” in Landscapes of leisure: Space, place and identities. eds. Gammon S. Elkington S. (London: Palgrave Macmillan UK), 135–146.

[ref17] Buckley R. (2006). Adventure tourism. Wallingford, UK: CABI.

[ref18] Buckley R. (2021). Is adventure tourism therapeutic? Tour. Recreat. Res. 46, 553–557. doi: 10.1080/02508281.2021.1931775

[ref19] Cater C. I. (2013). “The meaning of adventure” in Adventure tourism. eds. Taylor S. Varley P. Johnston T. (London: Routledge), 7–18.

[ref20] Clough P. Houge Mackenzie S. Mallabon L. Brymer E. (2016). Adventurous physical activity environments: a mainstream intervention for mental health. Sports Med. 46, 963–968. doi: 10.1007/s40279-016-0503-3, PMID: 26895993

[ref21] Davids K. Araújo D. Brymer E. (2016). Designing affordances for health-enhancing physical activity and exercise in sedentary individuals. Sports Med. 46, 933–938. doi: 10.1007/s40279-016-0511-3, PMID: 26914265

[ref22] Dobud W. W. Cavanaugh D. L. Harper N. J. (2020). Adventure therapy and routine outcome monitoring of treatment: the time is now. J. Exp. Educ. 43, 262–276. doi: 10.1177/1053825920911958

[ref23] Elsrud T. (2001). Risk creation in traveling: backpacker adventure narration. Ann. Tour. Res. 28, 597–617. doi: 10.1016/S0160-7383(00)00061-X

[ref24] Gibson J. J. (1979). The ecological approach to visual perception. Houghton: Houghton Lifflin.

[ref25] Harper N. J. Fernee C. R. Gabrielsen L. E. (2021). Nature’s role in outdoor therapies: an umbrella review. Int. J. Environ. Res. Public Health 18:5117. doi: 10.3390/ijerph18105117, PMID: 34065947 PMC8150931

[ref26] Immonen T. Brymer E. Davids K. Jaakkola T. (2022). An ecological dynamics approach to understanding human-environment interactions in the adventure sport context-implications for research and practice. Int. J. Environ. Res. Public Health 19:3691. doi: 10.3390/ijerph19063691, PMID: 35329384 PMC8954411

[ref27] Kerr J. H. Houge Mackenzie S. (2012). Multiple motives for participating in adventure sports. Psychol. Sport Exerc. 13, 649–657. doi: 10.1016/j.psychsport.2012.04.002

[ref28] King J. Hardwell A. Brymer E. Bedford A. (2020). Reconsidering McKenzie's six adventure education programming elements using an ecological dynamics lens and its implications for health and wellbeing. Sports 8:20. doi: 10.3390/sports8020020, PMID: 32054044 PMC7077236

[ref29] Li D. Newman G. Zhang T. Zhu R. Horney J. (2021). Coping with post-hurricane mental distress: the role of neighborhood green space. Soc. Sci. Med. 281:114084. doi: 10.1016/j.socscimed.2021.114084, PMID: 34107388 PMC8536201

[ref30] Monasterio E. Brymer E. (2015). “Mountaineering personality and risk” in Mountaineering tourism. eds. Musa G. Higham J. Thompson-Carr A. (New York: Routledge).

[ref31] Naidoo P. Ramseook-Munhurrun P. Seebaluck N. V. Janvier S. (2015). Investigating the motivation of baby boomers for adventure tourism. Procedia Soc. Behav. Sci. 175, 244–251. doi: 10.1016/j.sbspro.2015.01.1197

[ref32] Palmer C. (2002). ‘Shit happens’: the selling of risk in extreme sport. Aust. J. Anthropol. 13, 323–336. doi: 10.1111/j.1835-9310.2002.tb00213.x

[ref33] Peacock S. Brymer E. Davids K. Dillon M. (2017). An ecological dynamics perspective on adventure tourism. Tour. Rev. Int. 21, 307–316. doi: 10.3727/154427217X15022104437756

[ref34] Pizam A. Jeong G.-H. Reichel A. van Boemmel H. Lusson J. M. Steynberg L.. (2004). The relationship between risk-taking, sensation-seeking, and the tourist behavior of young adults: a cross-cultural study. J. Travel Res. 42, 251–260. doi: 10.1177/0047287503258837

[ref35] Pomfret G. Bramwell B. (2016). The characteristics and motivational decisions of outdoor adventure tourists: a review and analysis. Curr. Issue Tour. 19, 1447–1478. doi: 10.1080/13683500.2014.925430

[ref36] Pomfret G. Sand M. May C. (2023). Conceptualising the power of outdoor adventure activities for subjective well-being: a systematic literature review. J. Outdoor Recreat. Tour. 42:100641. doi: 10.1016/j.jort.2023.100641

[ref37] Rietveld E. Kiverstein J. (2014). A rich landscape of affordances. Ecol. Psychol. 26, 325–352. doi: 10.1080/10407413.2014.958035, PMID: 36152403

[ref38] Russell K. C. Gillis H. L. Hayes M. (2020). Adventure therapy treatment for young adult males struggling with addictions. J. Health Serv. Psychol. 46, 13–20. doi: 10.1007/s42843-020-00003-4

[ref39] Sharma-Brymer V. (2022). “Giving Back: an autoethnographic analysis of adventure experience as transformational” in Adventure psychology: Going knowingly into the unknown. eds. Reid P. Brymer E. (London: Routledge).

[ref40] Sharma-Brymer V. Brymer E. Davids K. (2015). The relationship between physical activity in green space and human health and wellbeing: an ecological dynamics perspective. Available at: http://www.joper.org/JOPER/JOPERVolume2_Issue1_1_3_2015_23.pdf

[ref41] Sharma-Brymer V. Brymer E. Mitra S. (2025). “Adventure tourism and tourist experience: revisiting diversity and inclusion” in Routledge handbook of adventure tourism. eds. Pomfret G. Doran A. Cater C. (London: Routledge).

[ref42] Stoffregen T. A. (2003). Affordances as properties of the animal-environment system. Ecol. Psychol. 15, 115–134. doi: 10.1207/S15326969ECO1502_2, PMID: 32803548

